# Supplementary material for: A global lipid map reveals host dependency factors conserved across SARS-CoV-2 variants
Source: Nat Commun. 2022 Jun 17;13:3487. doi: 10.1038/s41467-022-31097-7 (PMC9203258; doi:10.1038/s41467-022-31097-7)
Supplement: Supplementary file 1 — Supplementary Information [file 41467_2022_31097_MOESM1_ESM.pdf]

Supplementary Information

A global lipid map reveals host dependency factors conserved accross SARS-CoV-2 variants

Scotland E. Farley[1,4], Jennifer E. Kyle[2], Hans C. Leier[1], Lisa M. Bramer[3], Jules Weinstein[1], Timothy A. Bates[1], Joon-Yong Lee[2], Thomas O. Metz[2], Carsten Schultz[4], Fikadu G. Tafesse[1*]

[1]Department of Microbiology & Immunology, Oregon Health & Science University; Portland, OR, USA

[2]Department of Chemical Physiology and Biochemistry, Oregon Health & Science University; Portland, OR, USA

[3]Biological Sciences Division, Earth and Biological Sciences Directorate, Pacific Northwest National Laboratory (PNNL); Richland, WA, USA

[4]Computational Biology Group, Biological Sciences Division, Earth & Biological Systems Directorate, PNNL; Richland, WA, USA

* Correspondence and requests should be directed to FGT (email: tafesse@ohsu.edu)

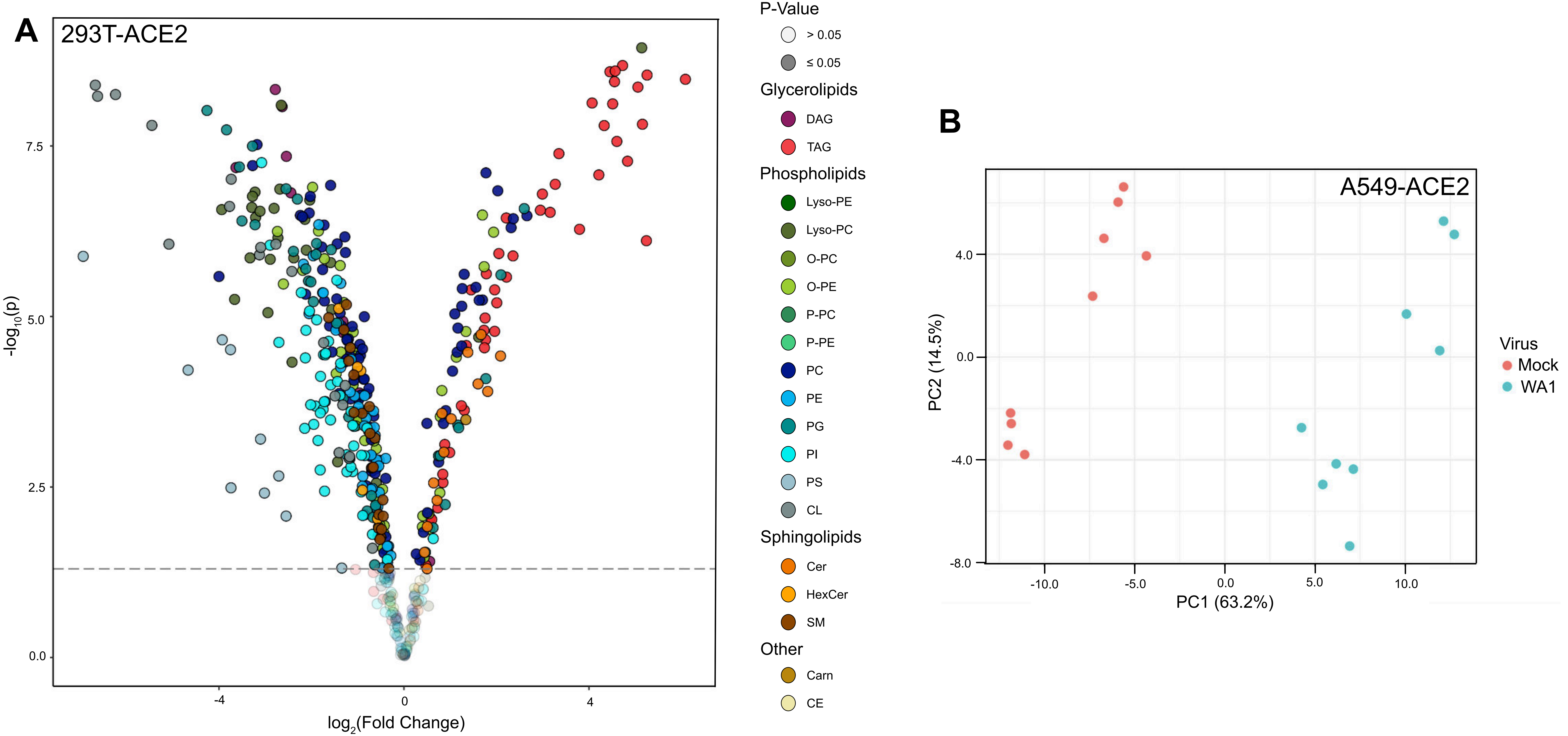

Supplementary Fig. 1 Further context for lipidomics datasets. (A) Volcano plot of all lipid species observed in the HEK293T-ACE2 live virus lipidomics dataset. Data points are means from 5 biological replicates; each data point represents a lipid species. P-values have been derived from a one-way ANOVA with Benjamini-Hochmini adjustment for multiple comparisons. DAG = diacylglycerol; TAG = triacylglycerol; Lyso-PC = lysophosphatidylcholine; Lyso-PE = lysophosphatidylcholine; O-PC = phosphatidylcholine (ether-linked); O-PE = phosphatidylethanolamine (ether-linked); P-PC = phosphatidylcholine (plasmologen-linked); P-PE = phosphatidylethanolamine (plasmologen-linked); PC = phosphatidylcholine; PE = phosphatdiylethanolamine; PG = phosphatidylglycerol; PI = phosphatidylinositol; PS = phosphatidylserine; CL = cardiolipin; Cer = ceramide; HexCer = hexosylceramide; SM = sphingomyelin; Carn = acylcarnitine; CE = cholesterol ester. (B) Principal component analysis of A549-ACE2 lipidomics experiment. Red dots are mock-infected dishes; blue dots are wild-type SARS-CoV-2 (WA1)-infected dishes.

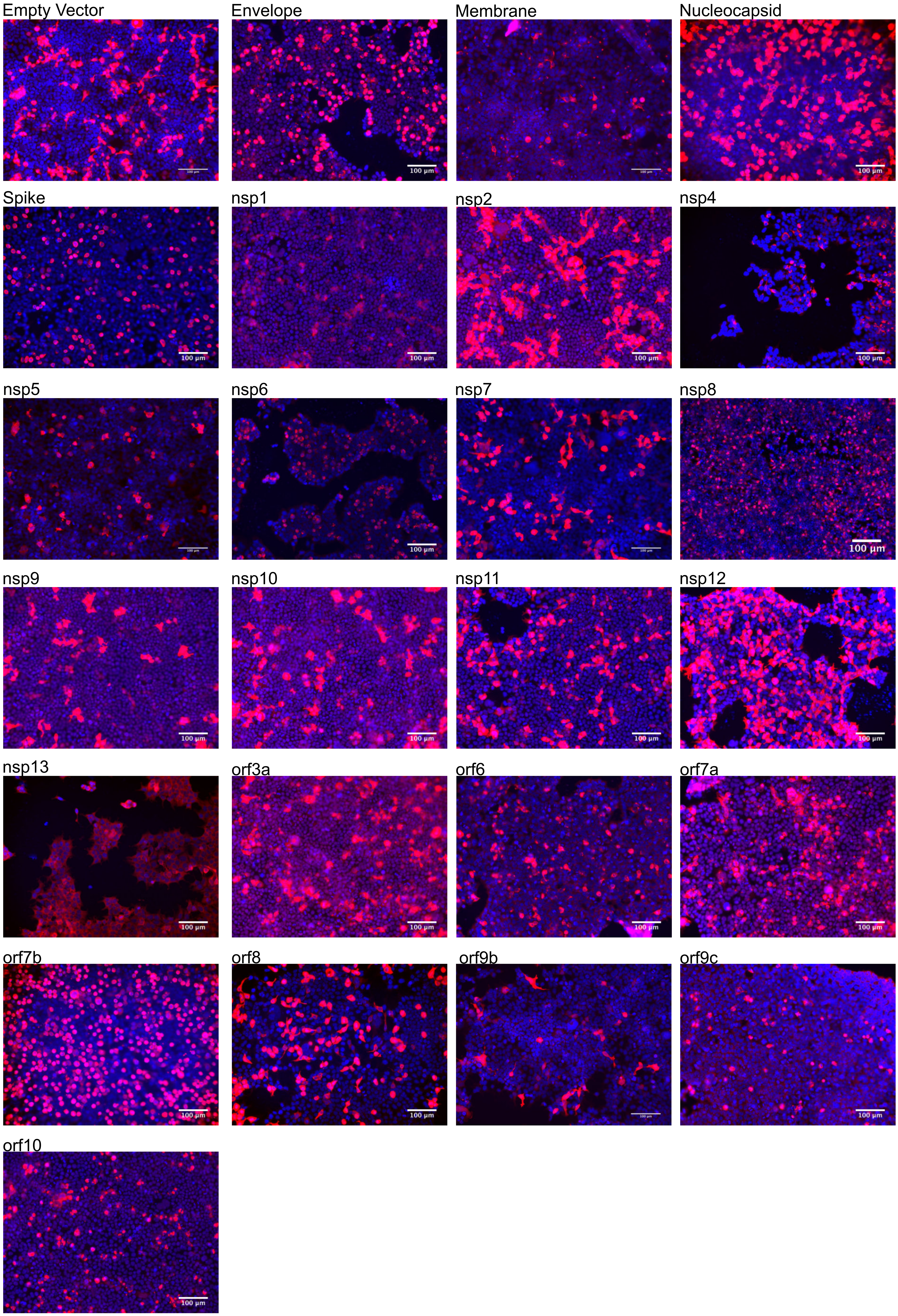

Supplementary Fig. 2 Images of HEK293T (wild type) cells transfected with individual viral proteins taken on a BZ-X700 all-in-one fluorescent microscope (Keyence) at 20x resolution. Red is anti-Strep-tag immunostaining; blue is DAPI. The amount of DNA used in each transfection can be found in Table S1. Images are representative of two independent experiments.

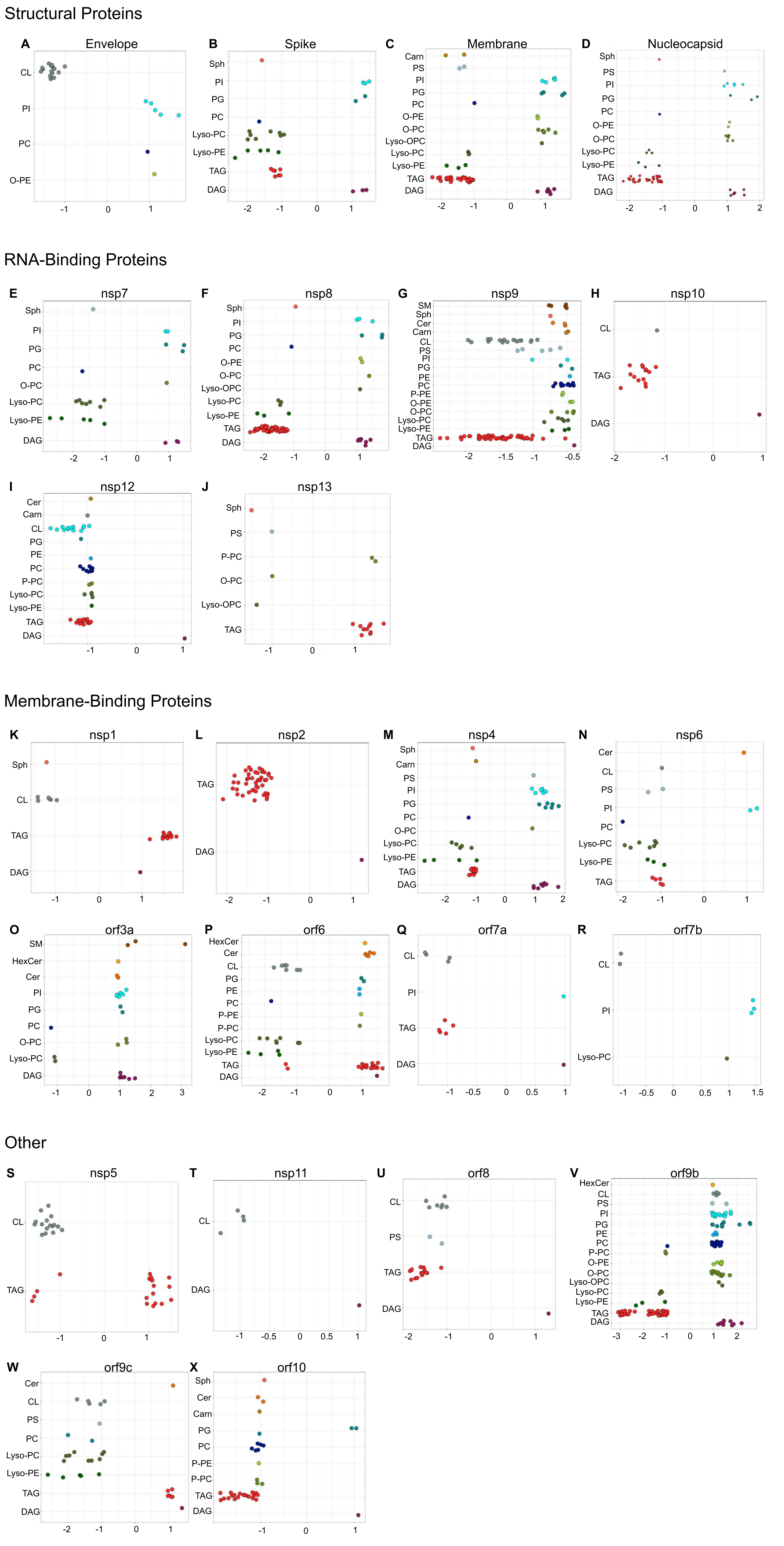

Supplementary Figure 3 For each viral protein, the lipids that changed significantly ($p < 0.05$, ANOVA, with Benjamini-Hochmini adjustment for multiple comparisons) relative to empty vector were selected. To identify the most influenced pathways, only lipids with a $\log_2$(fold change) greater than 0.9 or less than -0.9 were plotted. Log_2(fold change) relative to empty vector is shown on the x-axis.

(A-D) Structural proteins
(E-J) RNA-binding proteins
(K-R) Membrane-binding proteins
(S-X) Other proteins

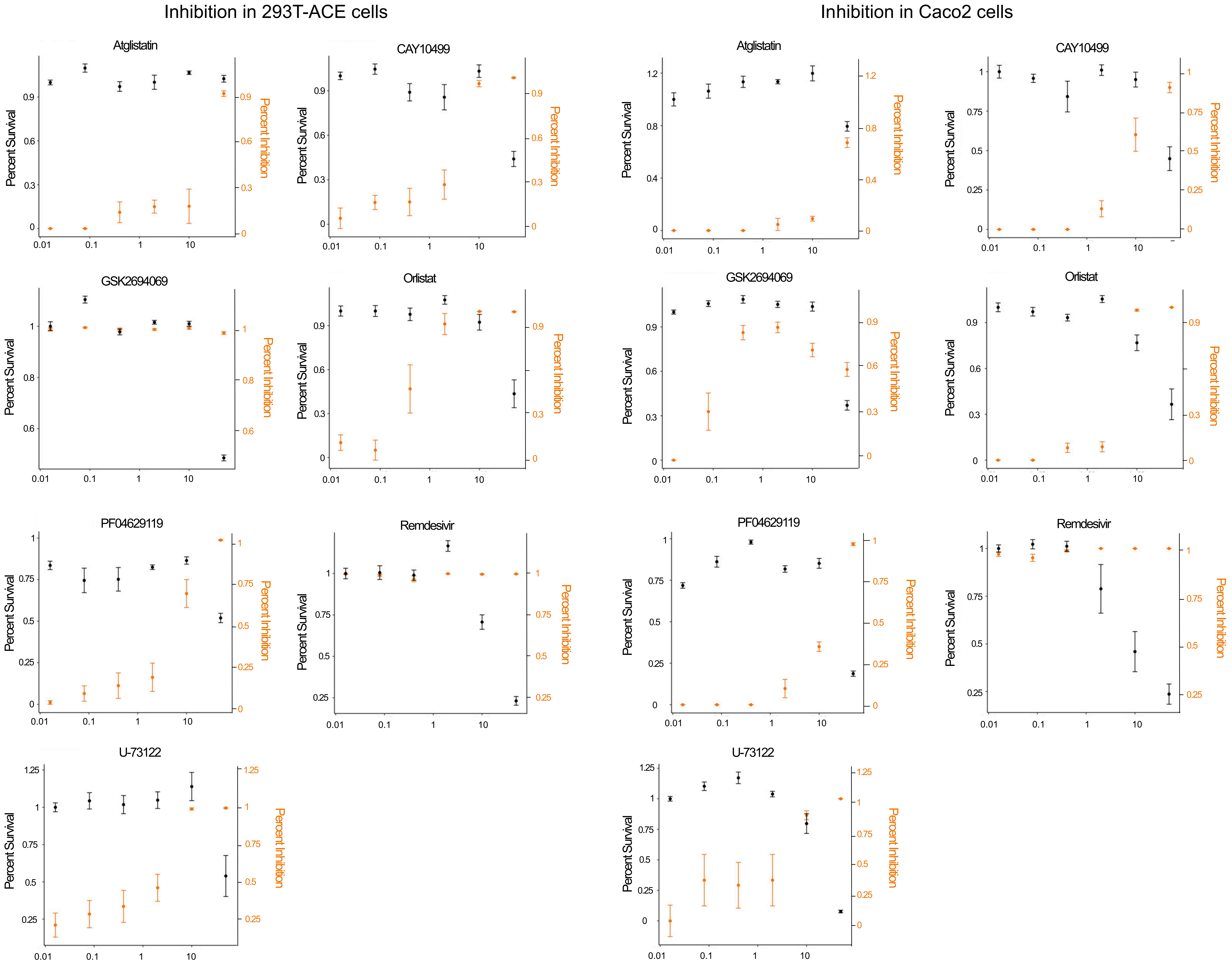

Supplementary Fig. 4 Compound cytotoxicity and inhibition of SARS-CoV-2 infection for relevant compounds. Compound concentrations (micromolar) are shown on the x-axis. "Percent survival" values are derived from three independent resazurin cytotoxicity experiments after 72 hours of compound treatment. 100% survival is defined as resazurin fluorescence in vehicle-treated cells. "Percent inhibition" value are derived from three independent focus forming assays after overnight compound treatment + 48 hours of infection. 100% infection is defined as the number of foci that result from vehicle-treated cells. Data are mean ± SE from three independent experiments.

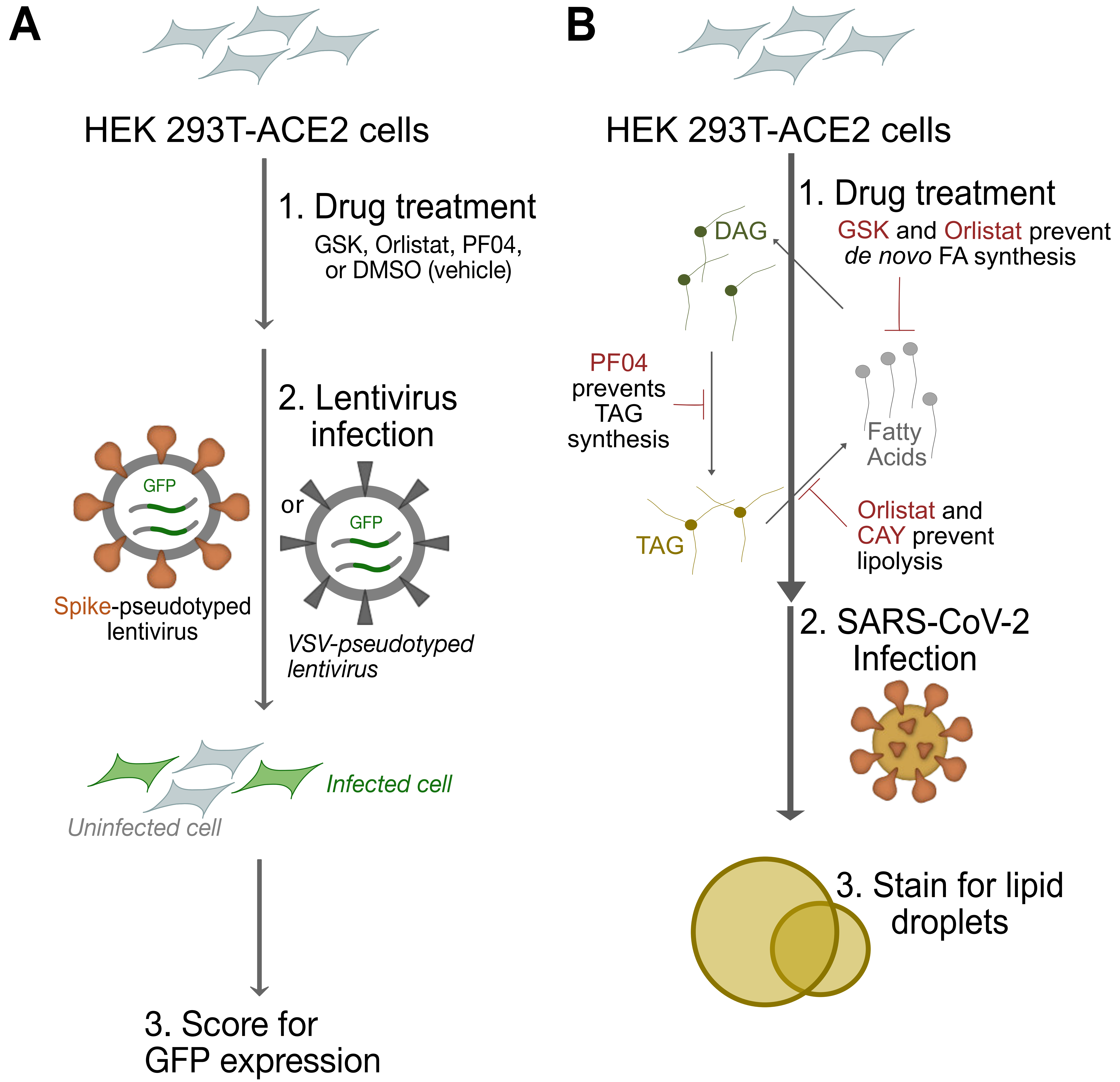

Supplementary Fig. 5 Description of assays used to validate inhibitor action. (**A**) SARS-CoV-2 entry assay using Spike-pseudotyped lentivirus bearing a GFP reporter, where Spike-pseudoviruses (or VSV G-pseudotyped control viruses) are used to infect HEK293T-ACE2 cells, and successful infections are quantified by scoring for GFP expression in high-content microscopy. (**B**) Assay to test the effect of glycerolipid inhibition on SARS-CoV-2 induced lipid droplet formation.

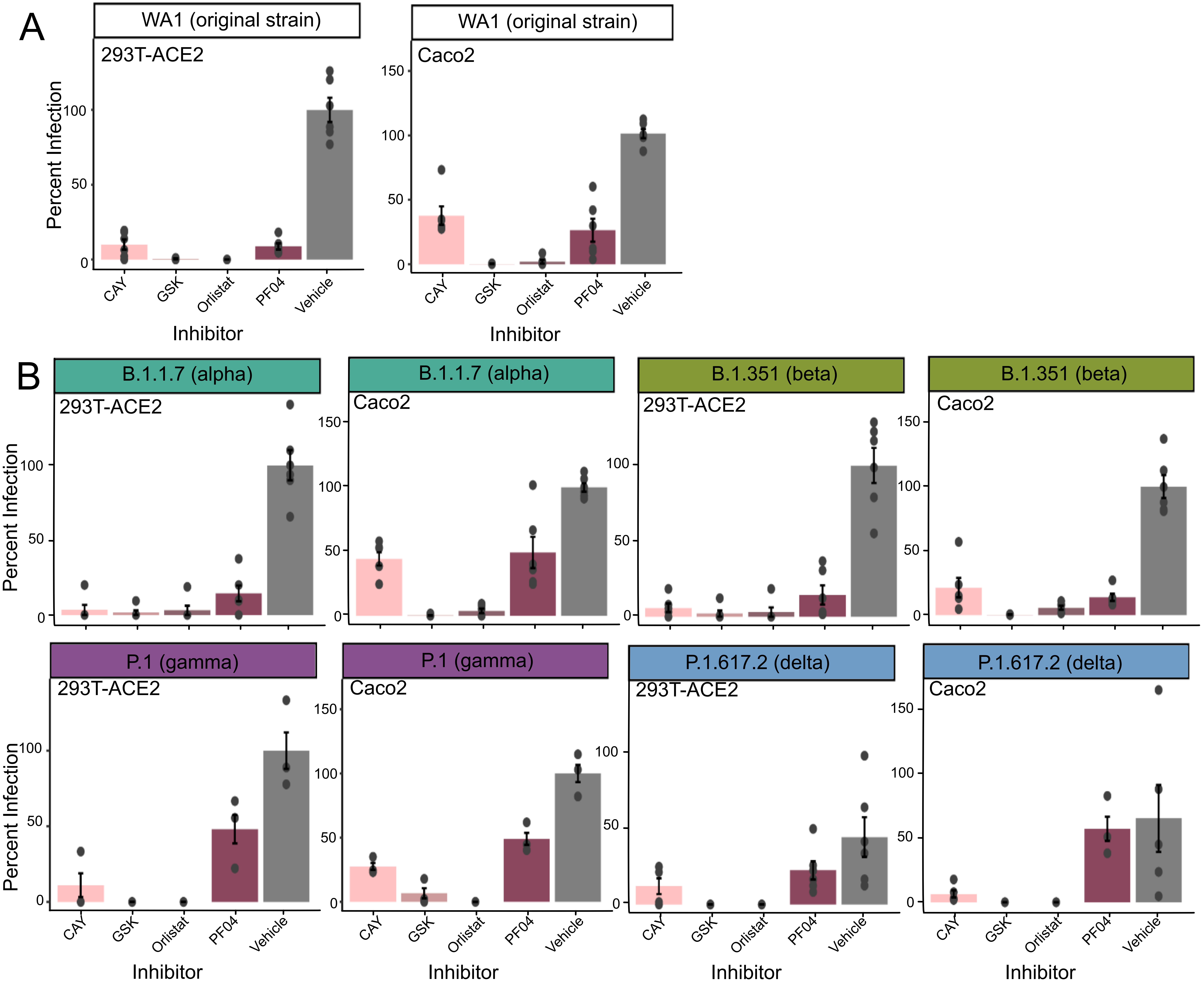

Supplementary Fig. 6 Inhibition of major variants of concern using glycerolipid biosynthesis inhibitors in two cell lines (all inhibitors used at 10 μM). Percent infection is defined as in supplemental Fig. 5. (**A**) Inhibitor efficacy against wild type (WA1) SARS-CoV-2 infection. (**B**) Inhibitor efficacy against major variants of concern. Data are from three independent experiments; data are mean ± SE for n = 6 biological replicates

Viral Protein	Plasmid ID	DNA amount, lipidomics (μg)	DNA amount, microscopy (μg)
nsp1	A01	30	3
nsp2	A02	30	3
nsp4	A05	30	3
nsp5	A06	30	3
nsp7	A09	20	2
nsp8	A10	30	3
nsp9	A11	40	4
nsp10	A12	35	3.5
nsp11	B01	30	3
nsp12	B02	45	4.5
nsp13	B03	35	3.5
S	B07	30	3
orf3a	B08	30	3
E	B10	30	3
M	B11	20	2
orf6	B12	35	3.5
orf7a	C01	35	3.5
orf7b	C02	30	3
orf8	C03	30	3
N	C04	10	1
orf9b	C05	30	3
orf9c	C06	35	3.5
orf10	C07	45	4.5
Empty Vector	PLVX	35	3.5

Supplementary Table 1. Amount of DNA used to transfect HEK293T and Caco2 cells in lipidomics experiments (related to Figure 2 and Figure 3) and microscopy experiments (related to figure 4).
