# Supplementary material for: A global lipid map reveals host dependency factors conserved across SARS-CoV-2 variants
Source: Nat Commun. 2022 Jun 17;13:3487. doi: 10.1038/s41467-022-31097-7 (PMC9203258; doi:10.1038/s41467-022-31097-7)
Supplement: Supplementary file 3 — Description of Additional Supplementary Information [file 41467_2022_31097_MOESM3_ESM.docx]

**Description of Additional Supplementary Information**

**File Name**: Supplementary Data 1

**Description:** Processed lipidomics data for live virus infection in HEK293T-ACE2 cells, related to Figure 1, Figure 3, and Supplementary Figure 1. Average fold changes and Benjamini-Hochmini adjusted p-values from one-way ANOVA tests are provided.

**File Name:** Supplementary Data 2

**Description:** Processed lipidomics data for live virus infection in A549-ACE2 cells, related to Figure 1 and Supplementary Figure 1. Average fold changes and Benjamini-Hochmini adjusted p-values from one-way ANOVA tests are provided.

**File Name**: Supplementary Data 3

**Description:** Processed lipidomics data for viral protein transfection in HEK293T cells, related to Figure 2, Figure 3, Supplementary Figure 2, and Supplementary Figure 3. Average fold changes and Benjamini-Hochmini adjusted p-values from one-way ANOVA tests are provided.
