# Supplementary material for: A global lipid map reveals host dependency factors conserved across SARS-CoV-2 variants
Source: Nat Commun. 2022 Jun 17;13:3487. doi: 10.1038/s41467-022-31097-7 (PMC9203258; doi:10.1038/s41467-022-31097-7)
Supplement: Supplementary file 7 — Reporting Summary [file 41467_2022_31097_MOESM7_ESM.pdf]

nature portfolio

Corresponding author(s): Fikadu Tafesse

Last updated by author(s): 2022.05.22

Reporting Summary

Nature Portfolio wishes to improve the reproducibility of the work that we publish. This form provides structure for consistency and transparency in reporting. For further information on Nature Portfolio policies, see our Editorial Policies and the Editorial Policy Checklist.

Statistics

For all statistical analyses, confirm that the following items are present in the figure legend, table legend, main text, or Methods section.

n/a	Confirmed	
☐	☒	The exact sample size (*n*) for each experimental group/condition, given as a discrete number and unit of measurement
☐	☒	A statement on whether measurements were taken from distinct samples or whether the same sample was measured repeatedly
☐	☒	The statistical test(s) used AND whether they are one- or two-sided<br>*Only common tests should be described solely by name; describe more complex techniques in the Methods section.*
☒	☐	A description of all covariates tested
☐	☒	A description of any assumptions or corrections, such as tests of normality and adjustment for multiple comparisons
☐	☒	A full description of the statistical parameters including central tendency (e.g. means) or other basic estimates (e.g. regression coefficient) AND variation (e.g. standard deviation) or associated estimates of uncertainty (e.g. confidence intervals)
☐	☒	For null hypothesis testing, the test statistic (e.g. *F*, *t*, *r*) with confidence intervals, effect sizes, degrees of freedom and *P* value noted<br>*Give P values as exact values whenever suitable.*
☒	☐	For Bayesian analysis, information on the choice of priors and Markov chain Monte Carlo settings
☒	☐	For hierarchical and complex designs, identification of the appropriate level for tests and full reporting of outcomes
☐	☒	Estimates of effect sizes (e.g. Cohen's *d*, Pearson's *r*), indicating how they were calculated

Our web collection on statistics for biologists contains articles on many of the points above.

Software and code

Policy information about availability of computer code

Data collection	All MS data was acquired on a Waters Aquity UPLS H class system interfaced with a Velos-ETD Orbitrap mass spectrometer
Data analysis	Lipidomics data were collected in positive and negative ionization modes and analyzed using R version 4.1.2. Images were processed and analyzed using CellProfiler 3.1.8. Foci in focus forming assays were counted using the Viridot R package.

For manuscripts utilizing custom algorithms or software that are central to the research but not yet described in published literature, software must be made available to editors and reviewers. We strongly encourage code deposition in a community repository (e.g. GitHub). See the Nature Portfolio guidelines for submitting code & software for further information.

Data

Policy information about availability of data

All manuscripts must include a data availability statement. This statement should provide the following information, where applicable:

- Accession codes, unique identifiers, or web links for publicly available datasets
- A description of any restrictions on data availability
- For clinical datasets or third party data, please ensure that the statement adheres to our policy

The raw lipidomics datasets generated during this study have been deposited and will be available at ftp://massive.ucsd.edu/MSV000087944/. Summaries of fold change changes and p-values are provided in Supplementary Data 1 (live virus lipidomics) and Supplementary Data 2 (viral protein lipidomics). Source data for other figures are provided for this paper.

Field-specific reporting

Please select the one below that is the best fit for your research. If you are not sure, read the appropriate sections before making your selection.

- [x] Life sciences
- [ ] Behavioural & social sciences
- [ ] Ecological, evolutionary & environmental sciences

For a reference copy of the document with all sections, see nature.com/documents/nr-reporting-summary-flat.pdf

Life sciences study design

All studies must disclose on these points even when the disclosure is negative.

Sample size	Sample sizes were determined based on previous experience with quantitative lipidomics, detailed microscopy image analysis and viral focus forming assays. We believe our sample sizes are sufficient based on our ability to draw strong statistical distinctions between different treatment groups.
Data exclusions	For our pseudovirus entry assay, there were five biological replicates for each condition, and the biggest outlier was removed from analysis due to inherent variability in the assay; we had established this criteria prior to performing the experiment as standard for this assay.
Replication	Reproducibility between bioreplicates in a lipidomics LC-MS/MS can be assessed by comparing the log2 abundances for each observed lipid in each replicate (n = 5). Inhibition experiments were performed independently three times to ensure reproducibility of results, and reproduction was successful. Microsocpy experiments were performed twice to ensure reproducibility of results, and reproduction was successful.
Randomization	Sample randomization is not relevant to our study. All experiments are based in cell culture, where samples already represent random dishes of cells
Blinding	Blinding is not relevant to LS-MS/MS data because data are acquired and processed systematically with established methods for lipid identification and quantification. The same is true for microscopy data, which was processed according to a universal pipeline where all images and conditions were treated identically, and focus forming assay data, in which 96-well plates were counted using the Viridot R package using the same settings for each plate of an experiment.

Reporting for specific materials, systems and methods

We require information from authors about some types of materials, experimental systems and methods used in many studies. Here, indicate whether each material, system or method listed is relevant to your study. If you are not sure if a list item applies to your research, read the appropriate section before selecting a response.

Materials & experimental systems

n/a	Involved in the study	
[ ]	[x]	Antibodies
[ ]	[x]	Eukaryotic cell lines
[x]	[ ]	Palaeontology and archaeology
[x]	[ ]	Animals and other organisms
[x]	[ ]	Human research participants
[x]	[ ]	Clinical data
[x]	[ ]	Dual use research of concern

Methods

n/a	Involved in the study	
[x]	[ ]	ChIP-seq
[x]	[ ]	Flow cytometry
[x]	[ ]	MRI-based neuroimaging

Antibodies

Antibodies used	anti-dsRNA, mouse IgG (Millipore, MABE1134; clone rJ2) (1:50 dilution)<br>anti-Strep-tag II, mouse IgG (Abcam, ab184224) (1:250 dilution)<br>anti-mouse IgG AlexaFluor647 (Invitrogen, A32728) (1:500 dilution)<br>Anti-llama secondary HRP, goat IgG (H + L) (Novus, NB7242) (1:5,000 dilution)
Validation	anti-dsRNA: evaluated by immunocytochemistry in Dengue virus infected A549 cells (manufacturer's website)<br>anti-Strep-tag II: immunofluorescence of the Strep tag only produces signal in Strep-tag-transfected cells<br>anti-mouse IgG AlexaFluor 647: only produces signal in cells stained with mouse primary antibodies; over 30 citations on manufacturer's website<br>anti-llama HRP: effectively stains alpaca-antibody-treated focus forming assays; cross-reactivity with human antigens is not observed

Eukaryotic cell lines

Policy information about cell lines

Cell line source(s)	HEK293T, Caco2, Vero E6 cell lines were obtained from ATCC (product numbersCRL-3216, HTB-37, and CRL-1586, respectively). 293T-ACE2 and A549-ACE2 cell lines were obtained from BEI resources (product numbers NR-52511 and NR-53821, respectively).
Authentication	All ATCC lines were already authenticated. BEI cell lines are produced by ATCC and are thus already authenticated by STR profiling.
Mycoplasma contamination	All cell lines tested negative for mycoplasma
Commonly misidentified lines (See ICLAC register)	No commonly misidentified lines were used in this study.
